# Within-Host and Population Transmission of *bla*_OXA-48_ in *K*. *pneumoniae* and *E*. *coli*

**DOI:** 10.1371/journal.pone.0140960

**Published:** 2015-10-20

**Authors:** Manon R. Haverkate, Mirjam J. D. Dautzenberg, Tjaco J. M. Ossewaarde, Anneke van der Zee, Jan G. den Hollander, Annet Troelstra, Marc J. M. Bonten, Martin C. J. Bootsma

**Affiliations:** 1 Julius Center for Health Sciences and Primary Care, University Medical Center Utrecht, Utrecht, the Netherlands; 2 Department of Medical Microbiology, University Medical Center Utrecht, Utrecht, the Netherlands; 3 Department of Medical Microbiology, Maasstad Ziekenhuis, Rotterdam, the Netherlands; 4 Department of Internal Medicine, Maasstad Ziekenhuis, Rotterdam, the Netherlands; 5 Department of Mathematics, Utrecht University, Utrecht, the Netherlands; University Medical Center Groningen, NETHERLANDS

## Abstract

During a large hospital outbreak of OXA-48 producing bacteria, most *K*. *pneumoniae*_OXA-48_ isolates were phenotypically resistant to meropenem or imipenem, whereas most *E*. *coli*_OXA-48_ isolates were phenotypically susceptible to these antibiotics. In the absence of molecular gene-detection *E*. *coli*_OXA-48_ could remain undetected, facilitating cross-transmission and horizontal gene transfer of *bla*_OXA-48_. Based on 868 longitudinal molecular microbiological screening results from patients carrying *K*. *pneumoniae*_OXA-48_ (n = 24), *E*. *coli*_OXA-48_ (n = 17), or both (n = 40) and mathematical modelling we determined mean durations of colonisation (278 and 225 days for *K*. *pneumoniae*_OXA-48_ and *E*. *coli*_OXA-48_, respectively), and horizontal gene transfer rates (0.0091/day from *K*. *pneumoniae* to *E*. *coli* and 0.0015/day vice versa). Based on these findings the maximum effect of horizontal gene transfer of *bla*_OXA-48_ originating from *E*. *coli*_OXA-48_ on the basic reproduction number (*R*_*0*_) is 1.9%, and it is, therefore, unlikely that phenotypically susceptible *E*. *coli*_OXA-48_ will contribute significantly to the spread of *bla*_OXA-48_.

## Introduction

Nosocomial outbreaks of carbapenemase-producing Enterobacteriaceae are rapidly increasing worldwide [1]. Three classes of carbapenemases have been identified in Enterobacteriaceae: Ambler class A (mainly *Klebsiella pneumoniae* carbapenemases), B (metallo-beta-lactamases), and D (oxacillinases) [2]. OXA-48, belonging to class D, was found for the first time in *K*. *pneumoniae* in 2001 in Turkey [3]. Since then, OXA-48 has been detected in many other countries [1,2,4–6], often due to transfer of patients from hospitals in OXA-48 endemic regions [2].

The *bla*_OXA-48_ gene is located on a plasmid [3] and spread of resistance can, therefore, occur through cross-transmission of bacteria (harbouring the plasmid), but also through within-host horizontal gene transfer (HGT) between the same and different species of Enterobacteriaceae, e.g., due to conjugation. Several studies have demonstrated that HGT, including of *bla*_OXA-48_, can be important in the transmission of highly resistant Enterobacteriaceae [7–12].

Based on international criteria for defining phenotypes based on antibiotic susceptibilities, OXA-48 producing bacteria can be highly resistant to carbapenems (mostly in *K*. *pneumoniae* and when present together with ESBL genes) or susceptible to imipenem and meropenem (e.g., in *Escherichia coli* in the absence of ESBL genes) [13]. In the latter case, identification of *bla*_OXA-48_ relies on molecular detection or susceptibility testing for ertapenem, which is not common practice in all diagnostic laboratories. Failure to identify these ‘susceptible’ OXA-48 producing isolates may facilitate unnoticed spread [2,12,14].

The dynamics of *bla*_OXA-48_ in Enterobacteriaceae are driven by the rates of bacterial cross-transmission, within-host HGT, and decolonisation rates. Yet, their values have never been quantified, although some studies suggest that the cross-transmission rate of *K*. *pneumoniae* is higher than of *E*. *coli* in hospital and household settings [15–17].

Data for this study were obtained from an outbreak in the Maasstad hospital, Rotterdam, the Netherlands, involving 118 patients [13]. Here we have used longitudinal colonisation data of 81 patients colonised with OXA-48 producing *K*. *pneumoniae* and/or *E*. *coli* of whom follow-up screening cultures were available and determined the within-host HGT-rate and duration of colonisation with OXA-48 producing bacteria. The outbreak was characterised by phenotypical resistance to carbapenems of 15.5% (meropenem) and 71.6% (imipenem) of *K*. *pneumoniae*_OXA-48_ and of 0% (meropenem) and 10.9% (imipenem) of *E*. *coli*_OXA-48_ using EUCAST breakpoints as determined by VITEK 2 (bioMérieux, Marcy l’Etoile, France). All tested isolates but 4 had an MIC ≥ 0.25 for ertapenem as determined by Etest (bioMérieux, Marcy l’Etoile, France) [13]. We, therefore, consider the outbreak to be caused by *K*. *pneumoniae*_OXA-48_ with a phenotype detectable by routine susceptibility testing or by *E*. *coli*_OXA-48_ with a susceptible phenotype that can only be detected by molecular testing or ertapenem susceptibility testing. Most *E*. *coli*_OXA-48_ were found when patients were residing at home, indicating that the hospital outbreak was mostly caused by *K*. *pneumoniae*_OXA-48_ with subsequent HGT.

Here, we assess the influence of *E*. *coli*_OXA-48_ on the epidemiology of *bla*_OXA-48_ using the measured duration of colonisation, the within-host HGT rates, and mathematical modelling. The effect is quantified by the influence of *E*. *coli*_OXA-48_ on the *R*_*0*_ of all OXA-48 producing Enterobacteriaceae. *R*_*0*_ is the basic reproduction number (the average number of secondary cases caused by one typical infected (colonised) individual, in a population consisting of susceptibles only [18]).

## Methods

All patients included in this study presumably acquired OXA-48 producing Enterobacteriaceae during a large nosocomial outbreak in the Netherlands from 2009 to 2011 [13]. The *bla*_OXA-48_ carrier status of colonised patients was determined every two months after hospital discharge, until six consecutive negative cultures with two months in-between cultures. There were no attempts for elimination of *bla*_OXA-48_ colonisation. Screening swabs from rectum, throat, and possible infection sites were inoculated overnight in broth containing ertapenem (0.125 mg/L). The specimens were then tested by PCR for *bla*_OXA-48_. Positive samples were inoculated on CRE (Oxoid Brilliance™ CRE Agar) and McConkey agar (Oxoid) and the presence of *bla*_OXA-48_ was reconfirmed by PCR in every morphologically different isolate [13]. Data for the current study was collected until March 2013. Data collection was not designed for research purposes but for clinical care. Patient information was anonymised and de-identified prior to analysis. The Medical Ethics Review Committee of the Maasstad Ziekenhuis determined that this study was exempted from evaluation with regard to the Dutch Medical Research Involving Subjects Act. Details on microbiological methods, outbreak management, and impact of infection control measures can be found in Dautzenberg et al [13].

### Model description

**Fig 1:**
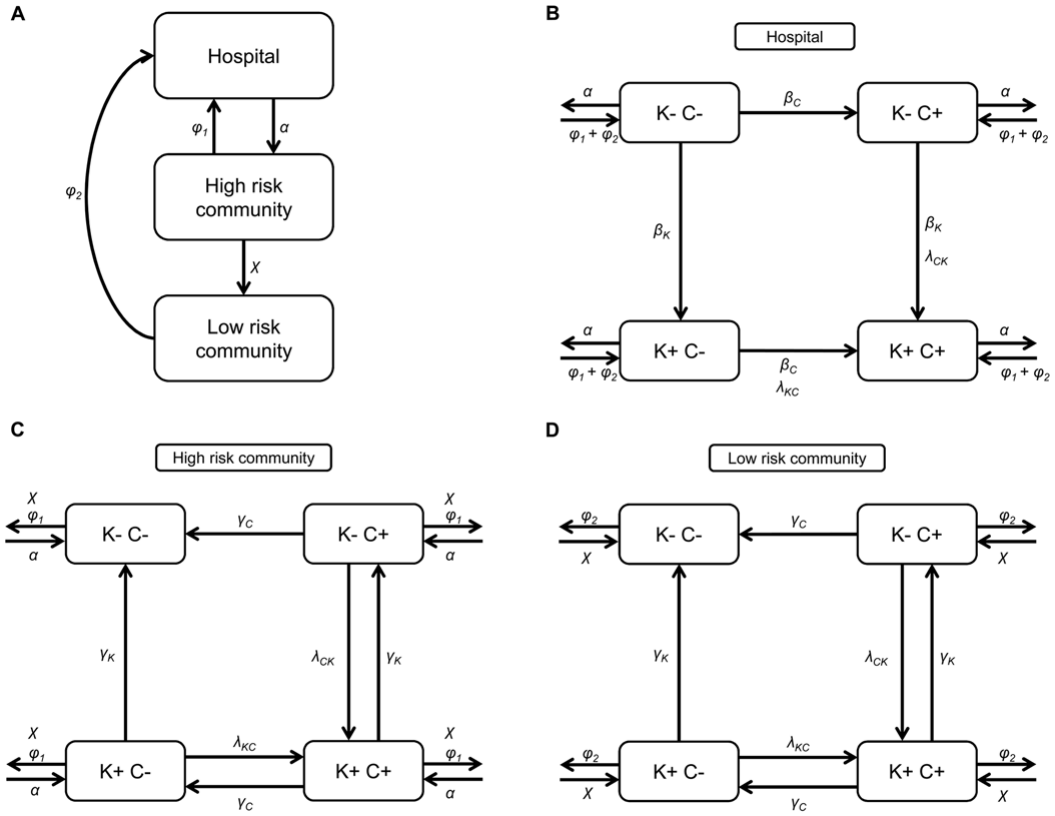
OXA-48 model. (a) Model of population flow. (b) Within-host model in the hospital. (c) Within-host model in the community with a high risk of readmission. (d) Within-host model in the community with a low risk of readmission. K- C-: *bla*_OXA-48_ negative (both *K*. *pneumoniae* and *E*. *coli* are susceptible). K- C+: *E*. *coli*_OXA-48_ (*K*. *pneumoniae* is susceptible). K+ C-: *K*. *pneumoniae*_OXA-48_ (*E*. *coli* is susceptible). K+ C+: *K*. *pneumoniae*_OXA-48_ and *E*. *coli*_OXA-48_.

We modelled a hospital and its corresponding catchment population with all subjects in either of four colonisation states: not colonised with OXA-48 producing *K*. *pneumoniae* or *E*. *coli* and susceptible to colonisation, colonised with *K*. *pneumoniae*_OXA-48_, colonised with *E*. *coli*_OXA-48_, or colonised with both *K*. *pneumoniae*_OXA-48_ and *E*. *coli*_OXA-48_ (Fig 1). All subjects were assumed to carry (antibiotic susceptible) *K*. *pneumoniae* and *E*. *coli*. We made no distinction between colonisation and infection. Cross-transmission was assumed to occur only during hospitalisation, at a rate proportional to the product of the number of *bla*_OXA-48_ colonised and susceptible patients according to the mass action principle. Within-host HGT of *bla*_OXA-48_ from *K*. *pneumoniae* to *E*. *coli* and vice versa was assumed to occur at a constant rate, both in- and outside the hospital setting. It was assumed that elimination of *bla*_OXA-48_ colonisation did not occur during hospitalisation (because of high antibiotic pressure at hospital level) and occurred at a fixed rate after hospital discharge.

When discharged from the hospital, patients entered the corresponding colonisation state in the community. As recently discharged patients have a higher risk of readmission, the readmission rate was modelled by dividing non-hospitalised subjects into two compartments. After discharge patients entered compartment 1 with a high readmission rate and with a fixed rate they moved into compartment 2, in which subjects had a lower readmission rate. Discharged patients were immediately replaced by new admissions with subjects from the community with either of four colonisation states, dependent on the prevalence of *bla*_OXA-48_ colonisation in the community [19]. Bed occupancy in the hospital was assumed to be constant (600 beds). All model parameters are listed in Table 1 and the differential equations are given in S1 File.

**Table 1:** Model parameters of the baseline model.

Parameter	Symbol	Default values
Mean length of stay	1/*α*	7 d
Cross-transmission rate *K*. *pneumoniae*_OXA-48_	*β*_*K*_	3 * *β*_*C*_, chosen to give *R*_*0*_ the desired value
Cross-transmission rate *E*. *coli*_OXA-48_	*β*_*C*_	Chosen to give *R*_*0*_ the desired value
Mean duration of colonisation *K*. *pneumoniae*_OXA-48_	1/*γ*_*K*_	278 d
Mean duration of colonisation *E*. *coli*_OXA-48_	1/*γ*_*C*_	225 d
HGT-rate *bla*_OXA-48_ from *K*. *pneumoniae* to *E*. *coli*	*λ*_*KC*_	0.0091 per day
HGT-rate *bla*_OXA-48_ from *E*. *coli* to *K*. *pneumoniae*	*λ*_*CK*_	0.0015 per day
Admission rate from high risk group	*φ*_*1*_	1/270 per day
Admission rate from low risk group	*φ*_*2*_	1/3897 per day, defined by other parameters: (*N α χ*) / (-*N α* + *M* (*φ*_*1*_ + *χ*)
Rate of change from high to low risk group	*χ*	1/100 per day
Hospital population	*N*	600
Catchment population (high + low risk)	*M*	250,000

HGT = horizontal gene transfer.

For given parameter values we can calculate the value of *R*_*0*_ in the model as the dominant eigenvalue of the next-generation matrix corresponding to the model [18] (see S1 File) as well as the per admission reproduction number *R*_*A*_ (the average number of *bla*_OXA-48_ transmission events due to one colonised individual during a single admission, i.e., ignoring the feedback loop due to readmission of patients who are still colonised) [19].

### Model parameterisation

The value of the discharge rate *α* was calculated as the reciprocal of the mean length of stay of all patients in the outbreak hospital, excluding admissions of one day.

Cross-transmission parameters for *K*. *pneumoniae*_OXA-48_ (*β*_*K*_) and *E*. *coli*_OXA-48_ (*β*_*C*_) have never been determined. However, to estimate the relative reduction of *R*_*0*_ due to interventions, only the ratio of values of the cross-transmission parameters, *β*_*K*_:*β*_*C*_, is important and not the absolute values. As the value of *R*_*0*_ for OXA-48 producing Enterobacteriaceae is unknown, we arbitrarily choose a value of *R*_*0*_ of 1.1 for our graphs. A ratio of 3:1 for *β*_*K*_:*β*_*C*_ was taken, based on studies that suggest that ESBL-producing *K*. *pneumoniae* spreads better than *E*. *coli* [15–17].

Decolonisation rates (*γ*_*K*_ and *γ*_*C*_) were calculated from the data of all patients who had at least one screening culture with *K*. *pneumoniae*_OXA-48_ or *E*. *coli*_OXA-48_ obtained since June 2011. It was assumed that patients were positive at least until their last positive test. Negative culture results in-between positive results were considered false negative. Negative cultures after the last positive culture could be true negative or false negative. The specificity was assumed to be 100% [13]. We jointly estimated the sensitivity of the detection method (swabbing, culturing, and PCR) and the parameter of the (assumed) exponentially distributed survival curve of *bla*_OXA-48_ colonisation with a maximum likelihood method. 95% confidence intervals (CI) were obtained using the likelihood ratio test (S2 File).

The HGT-rates *λ*_*KC*_ and *λ*_*CK*_ were also estimated from the results of the *bla*_OXA-48_ screening cultures, excluding patients in which the first positive screening culture yielded both *K*. *pneumoniae*_OXA-48_ and *E*. *coli*_OXA-48_. The HGT-rate of *bla*_OXA-48_ from *K*. *pneumoniae* to *E*. *coli* (*λ*_*KC*_) was determined for all patients with at least one positive screening culture for *K*. *pneumoniae*_OXA-48_ and without a previous culture with *E*. *coli*_OXA-48_, with in the denominator the number of days from first detection of *K*. *pneumoniae*_OXA-48_ to either the last culture date with *K*. *pneumoniae*_OXA-48_ or the middle date between the last culture date with *K*. *pneumoniae*_OXA-48_ and the first detection of *E*. *coli*_OXA-48_ and *K*. *pneumoniae*_OXA-48_. In the numerator we included all events in which *K*. *pneumoniae*_OXA-48_ colonisation preceded combined colonisation with *K*. *pneumoniae*_OXA-48_ and *E*. *coli*_OXA-48_. The HGT-rate was thus calculated as the number of transfers per day at risk for HGT. The HGT-rate *λ*_*CK*_ was calculated analogously. In this way, an effective HGT-rate in patients was estimated, providing an averaged rate that incorporates the influence of factors changing over time such as antibiotic use.

The estimates for the mean time to readmission from the population with a high risk of readmission (1/*φ*_*1*_) and the rate of moving from the population with a high risk of readmission to the population with a lower risk (*χ*) were obtained from real life data of the University Medical Center Utrecht [20]. The mean time to readmission from the population with a lower risk (1/*φ*_*2*_) was defined by the other parameters in the model, in order to keep population sizes in agreement with the data.

Population sizes in hospital and community were based on those of the Maasstad hospital, which contained approximately 600 beds and had a catchment area of approximately 250,000 people [21]. The sizes of the sub communities (high and lower risk of readmission) were defined by the model.

### Model calculations

The ratio between *R*_*0*_ and *R*_*A*_ indicates the relative importance of readmission in the nosocomial dynamics of *bla*_OXA-48_ spread.

The impact of HGT was measured by comparing the mean duration of colonisation with *K*. *pneumoniae*_OXA-48_ and *E*. *coli*_OXA-48_ as calculated from the data and model. As the gene can be exchanged between both species, HGT effectively prolongs the duration of colonisation. The exact derivation can be found in S1 File.

In theory, earlier detection of *E*. *coli*_OXA-48_ (e.g., by ertapenem susceptibility testing and molecular testing) would reduce nosocomial transmission of OXA-48 producing Enterobacteriaceae and the maximum effect would be comparable to immediate elimination of *E*. *coli*_OXA-48_. This theoretical effect was quantified by the relative difference between *R*_*0*_ with and without *E*. *coli*. Arbitrarily, a 20% reduction in *R*_*0*_ was considered effective.

Sensitivity analyses were performed for cross-transmission rates (*β*_*K*_ and *β*_*C*_), HGT-rates (*λ*_*KC*_ and *λ*_*CK*_), duration of colonisation (*γ*_*K*_ and *γ*_*C*_), and the catchment population size (*M*). Furthermore, we determined the potential effects of *bla*_OXA-48_ cross-transmission in the community and of different definitions for colonisation.

Analyses were done in SPSS (IBM SPSS Statistics, Version 20) and Mathematica (Wolfram Research, Inc., Mathematica, Version 9.0, Champaign, IL).

## Results

Duration of colonisation and HGT-rates were derived from longitudinal culture results of 81 patients; 24 carried *K*. *pneumoniae*_OXA-48_ only, 17 carried *E*. *coli*_OXA-48_ only, and 40 carried both *K*. *pneumoniae*_OXA-48_ and *E*. *coli*_OXA-48_ (Table 2). The estimated mean duration of colonisation was 278 (95% CI 204–394) and 225 days (95% CI 164–321) for *K*. *pneumoniae*_OXA-48_ and *E*. *coli*_OXA-48_, respectively.

**Table 2:** Baseline characteristics of the study population of positive patients (n = 81).

Gender, n males (%)	49 (60.5%)
Age at first admission during outbreak period, median (IQR)	69.1 (57.8–77.5)
Length of stay in days during outbreak period, median (IQR) per admission, excluding 1-day admissions	8 (3–18)
Admission days during outbreak period, median (IQR)	38 (14–68.5)
Number of admissions during outbreak period, median (IQR)	3.5 (2–6)
Number of days on which screening cultures were taken, median (IQR)	11 (8–15)
Number of days between two subsequent screening cultures, median (IQR)	18 (2–63)
Total number of screening cultures included, n	868
Screening cultures taken during hospital stay, %	41%
Time of follow-up since first positive culture until last known culture in days, mean (SD)	374 (175)
Patients at risk for HGT from *K*. *pneumoniae* to *E*. *coli*	47
Patients at risk for HGT from *E*. *coli* to *K*. *pneumoniae*	22

IQR = interquartile range.

SD = standard deviation.

HGT = horizontal gene transfer.

We observed 21 *bla*_OXA-48_ transfer events from *K*. *pneumoniae* to *E*. *coli* during 2305 days at risk, yielding a HGT-rate of 0.0091/day. There were three *bla*_OXA-48_ transfer events from *E*. *coli* to *K*. *pneumoniae* during 2004 days at risk, yielding a HGT-rate of 0.0015/day. HGT prolonged the mean duration of *bla*_OXA-48_ colonisation with a factor 1.2 and 1.3 for *K*. *pneumoniae*_OXA-48_ and *E*. *coli*_OXA-48_, respectively.

The sensitivities of the screening process for detecting *K*. *pneumoniae*_OXA-48_ and *E*. *coli*_OXA-48_ were estimated to be 90.0% (95% CI 86.1–93.3) and 89.7% (95% CI 84.4–93.8), respectively.

The ratio between *R*_*0*_ and *R*_*A*_ was 1.36, implying that readmissions of colonised patients were responsible for a more than a quarter of all transmissions (0.36/1.36 = 26%). As can be seen from S1 File, this ratio is dependent on the choice of the parameter values. However, it does not depend on the values for *β*_*K*_ and *β*_*C*_ but on the ratio of *β*_*K*_:*β*_*C*_.

**Fig 2:**
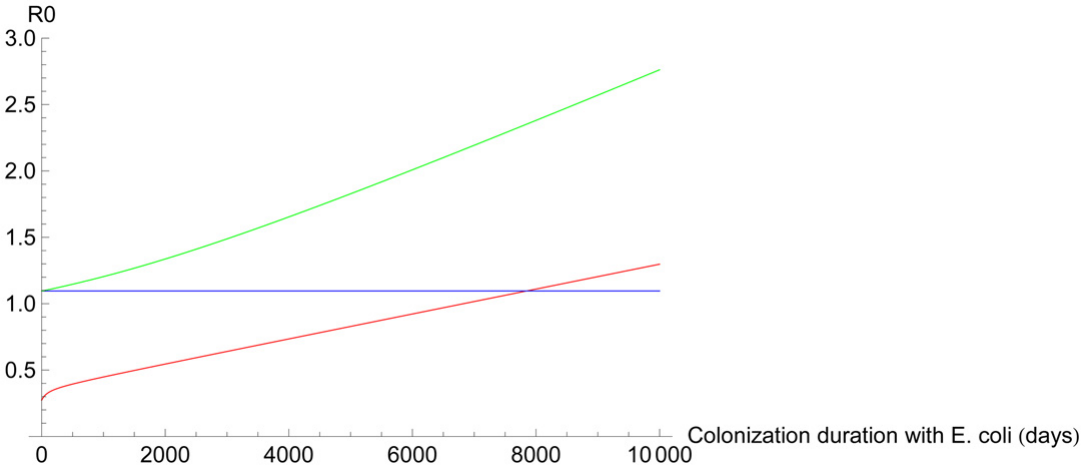
Influence of within-host horizontal gene transfer on *R*_*0*_. The red line depicts the *R*_*0*_ of *E*. *coli*_OXA-48_ as a function of the duration of colonisation with *E*. *coli*_OXA-48_ when there is no HGT, the blue line represents the *R*_*0*_ of *K*. *pneumoniae*_OXA-48_ as a function of the duration of colonisation with *E*. *coli*_OXA-48_ when there is no HGT, and the green line represents *R*_*0*_ as a function of the duration of colonisation with *E*. *coli*_OXA-48_ when there is HGT. *R*_*0*_ is arbitrarily set at 1.1.

The contribution of *E*. *coli*_OXA-48_ to *R*_*0*_ was 1.9% with current parameter values. A 20% contribution to *R*_*0*_ would be reached when the mean duration of colonisation with *E*. *coli*_OXA-48_ would be 6.1 years (instead of 225 days as observed) or the HGT-rates from *K*. *pneumoniae* to *E*. *coli* and vice versa would be 7.3 times the observed rates (Fig 2 and Table 3).

**Table 3:** Results sensitivity analyses on *β*_*K*_ and *β*_*C*_, all other parameters are kept constant.

	*β*_*K*_ = 3 *β*_*C*_ (baseline)	*β*_*K*_ = 6 *β*_*C*_	*β*_*K*_ = *β*_*C*_	*β*_*K*_ = 0.5 *β*_*C*_
Ratio *R*_*0*_/*R*_*A*_	1.36	1.36	1.45	1.33
Contribution of *E*. *coli*_OXA-48_ to *R*_*0*_	-1.9%	-1.6%	-9.8%	-50.1%
Duration of colonisation *E*. *coli* to reach 20% contribution of *E*. *coli*_OXA-48_ to *R*_*0*_ (days)	2228	2807	535	*
Ratio HGT-rates *K*. *pneumoniae*_OXA-48_ to *E*. *coli*_OXA-48_ and v.v. versus baseline values to reach 20% contribution of *E*. *coli*_OXA-48_ to *R*_*0*_	7.3	9.0	2.4	*

* 20% not achievable with this ratio of *β*_*K*_:*β*_*C*_.

HGT = horizontal gene transfer.

### Sensitivity analysis

Increasing the ratio between the cross-transmission parameters *β*_*K*_ and *β*_*C*_ only has a small effect on the contribution of *E*. *coli*_OXA-48_ to *R*_*0*_. The opposite, though, when the *β*_*K*_:*β*_*C*_ ratio declines, will increase the contribution of *E*. *coli*_OXA-48_, although this scenario does not seem realistic (Table 3).

Changing HGT-rates and duration of colonisation also influences results, but only to a small extent. Doubling both HGT-rates changes the effect of *E*. *coli*_OXA-48_ on *R*_*0*_ to 5.0%, while doubling both the duration of colonisation with *K*. *pneumoniae*_OXA-48_ and *E*. *coli*_OXA-48_ changes the effect to 7.0% (S3 File). Changes in the catchment population size (default +/- 100,000) do not impact the results.

In a setting with 20% of all *bla*_OXA-48_ cross-transmissions occurring in the community, while keeping the ratio of *β*_*K*_:*β*_*C*_ at 3:1, the contribution of *E*. *coli*_*OXA-48*_ to *R*_*0*_ was 2.6%. The duration of colonisation with *E*. *coli*_*OXA-48*_ should then be 3.3 years to reach a 20% contribution to *R*_*0*_ (S3 File).

Finally, we investigated the effects of different definitions for colonisation. In the default we only used negative culture results to define negativity. In this sensitivity analysis, a patient was considered non-colonised with *K*. *pneumoniae*_OXA-48_ when, on a certain day, *E*. *coli*_OXA-48_ but not *K*. *pneumoniae*_OXA-48_ was detected (and vice versa for *E*. *coli*). Using this definition, the mean durations of colonisation were still comparable to the baseline situation: 269 days for *K*. *pneumoniae*_OXA-48_ (95% CI 197–381) and 227 days for *E*. *coli*_OXA-48_ (95% CI 165–324). The sensitivity of the screening process for detecting *K*. *pneumoniae*_OXA-48_ then became 79.7% (95% CI 75.1–83.8) and for *E*. *coli*_OXA-48_ 74.8% (95% CI 68.7–80.4).

## Discussion

The combination of detailed microbiological data on duration of colonisation of *K*. *pneumoniae*_OXA-48_ and *E*. *coli*_OXA-48_, the estimated occurrence of within-host HGT between both species, and a mathematical model demonstrated that the feared scenario of a hidden reservoir of *bla*_OXA-48_ containing, but phenotypically carbapenem-susceptible, *E*. *coli* is highly unlikely. Based on the parameters derived during a large hospital outbreak of *K*. *pneumoniae*_OXA-48_, *bla*_OXA-48_ in phenotypically susceptible *E*. *coli* had minor effects on the *R*_*0*_ of OXA-48 containing Enterobacteriaceae. Furthermore, the model illustrates the strain characteristics needed, related to duration of colonisation and incidence of HGT, allowing unnoticed spread of *bla*_OXA-48_. In the current study the durations of colonisation were comparable for *K*. *pneumoniae*_OXA-48_ and *E*. *coli*_OXA-48_, but the HGT-rate of *bla*_OXA-48_ from *K*. *pneumoniae* to *E*. *coli* appeared much higher than the other way around.

Naturally, a model is a simplification of reality and model predictions should be interpreted with caution. For example, the population is considered to be homogeneous, so no distinction is made between different types of wards and patients. Moreover, parameter estimates are based on a single outbreak, reducing the external validity. No data was available on antibiotic use during the follow-up period, impairing estimates on the effect of antibiotics on OXA-48 producing bacteria. Yet, since there are no other studies with a similar data on *bla*_OXA-48_ transmission, we consider these outbreak data currently as the best available. Furthermore, only *K*. *pneumoniae* and *E*. *coli* are included in this model. Although other bacteria can harbour the *bla*_OXA-48_ plasmid, during this outbreak only 2.5% of all patients carrying OXA-48 producing bacteria did not carry *K*. *pneumoniae*_OXA-48_ or *E*. *coli*_OXA-48_ [13].

We did not distinguish colonisation from infection, although infected patients might have had a longer length of stay or higher transmission potential. Also, all different routes of transmission, such as direct patient to patient contact, transmission via health care workers, or transmission from environmental sources were captured in one transmission parameter. This seems justified since no colonisation was demonstrated in staff [13] but transient colonisation is still possible. Furthermore, when the duration of carriage in healthcare personnel is short, there is no need to model the process as vector-borne transmission [22]. In sensitivity analyses, the ratio of the transmission parameters for *K*. *pneumoniae*_OXA-48_ and *E*. *coli*_OXA-48_ was changed in order to investigate effects on the outcomes. Results would alter dramatically if the transmission parameter for *E*. *coli* would be higher than for *K*. *pneumoniae*. However, although there are no accurate estimates available, there is evidence that *K*. *pneumoniae* spreads better than *E*. *coli* and the ratio of 3:1 seems plausible [13,16,17]. Furthermore, allowing cross-transmission in the community increased the effect of *E*. *coli*_OXA-48_ only slightly. Also, 95% of *bla*_OXA-48_ encoding plasmids were identical in pMLST [13]. This strengthens our assumptions on cross-transmission and HGT. The assumption that all patients carry susceptible *E*. *coli* and *K*. *pneumoniae* might seem stringent, but the model estimates an effective HGT-rate. The assumption that all patients carry susceptible *K*. *pneumoniae* provides the most conservative estimate, since the rate will be higher in patients with susceptible Enterobacteriaceae and 0 in patients without. If we would assume that only 50% of the patients carries antibiotic-susceptible *K*. *pneumoniae*, the rate will be double (and the role of *E*. *coli* will likewise decline). When the populations are well mixed, the estimates, as determined by the model, will represent the effective (averaged) HGT-rates.

Within-host HGT prolongs the duration of colonisation. ‘Temporary storage’ of *bla*_OXA-48_ in another species, which may not be detected on its phenotypical appearance, can thus artificially prolong the duration of colonisation. However, the effect on transmission will be limited as long as most of the prolongation will occur outside the hospital where cross-transmission is assumed to occur less frequently than in health care settings. Since patients resided outside of the hospital during the follow-up period, we are confident that events we marked as HGT in this period were indeed HGT and not transmission, although strain relatedness was not assessed.

Even though transmission parameters and *R*_*0*_ of OXA-48 producing Enterobacteriaceae have never been quantified, we determined that readmission of colonised patients was important in the nosocomial dynamics of *bla*_OXA-48_ during this outbreak. Readmission of colonised patients may lead to new events of transmission creating a so-called ‘feedback loop’ in the dynamics [19]. We estimated that more than a quarter of transmissions were caused by readmitted patients during this outbreak, supporting the policy to flag colonised patients and to treat them in isolation or with barrier precautions when readmitted. In analogy of Cooper et al. [19] we emphasise the importance of readmission. Interventions should not only focus on reducing *R*_*A*_ below one, but also on getting *R*_*0*_ below one.

Although others have reported sensitivities of (nearly) 100% for PCR for *bla*_OXA-48_ [23–25], we estimated that the sensitivity of the screening procedures used during this outbreak was around 90%. However, this includes the procedure of obtaining and transporting swabs, overnight broth inoculation with ertapenem, PCR testing, and microbiological culture. While the analytical sensitivity of the PCR is probably close to 100%, each step might have yielded false negative results, reducing the sensitivity of the total procedure.

During the time of the outbreak (2011), screening for OXA-48 with ertapenem was not widely used, but the molecular screening on OXA-48 identified *E*. *coli*_OXA-48_ with low MICs for imipenem and meropenem instead. Our findings suggest that a failure to detect *E*. *coli*_OXA-48_ (if it had happened) would not have led to massive transmission of OXA-48 originating from undetected *E*. *coli*_OXA-48_ isolates. The model, although a simplification of reality, estimated that the contribution of *E*. *coli*_OXA-48_ to *R*_*0*_ during this OXA-48 outbreak was 1.9%, which suggests that it is unlikely that a large reservoir of *E*. *coli*_OXA-48_ will emerge if these isolates would remain undetected.

## Supporting Information

S1 File Calculations. (PDF)

S2 File Parametric and non-parametric estimation of the survival curve. (PDF)

S3 File Sensitivity analyses. (PDF)

## References

[1] Canton R, Akova M, Carmeli Y, Giske CG, Glupczynski Y, Gniadkowski M, et al Rapid evolution and spread of carbapenemases among Enterobacteriaceae in Europe. Clin Microbiol Infect. 2012; 18: 413–431. 10.1111/j.1469-0691.2012.03821.x 22507109

[2] Nordmann P, Naas T, Poirel L. Global spread of Carbapenemase-producing Enterobacteriaceae. Emerg Infect Dis. 2011; 17: 1791–1798. 10.3201/eid1710.110655 22000347 PMC3310682

[3] Poirel L, Heritier C, Tolun V, Nordmann P. Emergence of oxacillinase-mediated resistance to imipenem in Klebsiella pneumoniae. Antimicrob Agents Chemother. 2004; 48: 15–22. 14693513 10.1128/AAC.48.1.15-22.2004 PMC310167

[4] Potron A, Kalpoe J, Poirel L, Nordmann P. European dissemination of a single OXA-48-producing Klebsiella pneumoniae clone. Clin Microbiol Infect. 2011; 17: E24–E26. 10.1111/j.1469-0691.2011.03669.x 21973185

[5] Glupczynski Y, Huang TD, Bouchahrouf W, Redenze de Castro R, Bauraing C, Gérard M, et al Rapid emergence and spread of OXA-48-producing carbapenem-resistant Enterobacteriaceae isolates in Belgian hospitals. Int J Antimicrob Agents. 2012; 39: 168–172. 10.1016/j.ijantimicag.2011.10.005 22115539

[6] Kalpoe JS, Al Naiemi N, Poirel L, Nordmann P. Detection of an Ambler class D OXA-48-type beta-lactamase in a Klebsiella pneumoniae strain in The Netherlands. J Med Microbiol. 2011; 60: 677–678. 10.1099/jmm.0.028308-0 21252273

[7] Doi Y, Adams-Haduch JM, Peleg AY, D'Agata EM. The role of horizontal gene transfer in the dissemination of extended-spectrum beta-lactamase-producing Escherichia coli and Klebsiella pneumoniae isolates in an endemic setting. Diagn Microbiol Infect Dis. 2012; 74: 34–38. 10.1016/j.diagmicrobio.2012.05.020 22722012 PMC3427399

[8] Mooij MJ, Willemsen I, Lobbrecht M, Vandenbroucke-Grauls C, Kluytmans J, Savelkoul PH. Integron class 1 reservoir among highly resistant gram-negative microorganisms recovered at a Dutch teaching hospital. Infect Control Hosp Epidemiol. 2009; 30: 1015–1018. 10.1086/606039 19719415

[9] Nijssen S, Florijn A, Top J, Willems R, Fluit A, Bonten M. Unnoticed spread of integron-carrying Enterobacteriaceae in intensive care units. Clin Infect Dis. 2005; 41: 1–9. 15937755 10.1086/430711

[10] Norrby SR. Integrons: adding another threat to the use of antibiotic therapy. Clin Infect Dis. 2005; 41: 10–11. 15937756 10.1086/430715

[11] Crémet L, Bourigault C, Lepelletier D, Guillouzouic A, Juvin ME, Reynaud A, et al Nosocomial outbreak of carbapenem-resistant Enterobacter cloacae highlighting the interspecies transferability of the blaOXA-48 gene in the gut flora. J Antimicrob Chemother. 2012; 67: 1041–1043. 10.1093/jac/dkr547 22223227

[12] Mathers AJ, Cox HL, Kitchel B, Bonatti H, Brassinga AK, Carroll J, et al Molecular dissection of an outbreak of carbapenem-resistant enterobacteriaceae reveals Intergenus KPC carbapenemase transmission through a promiscuous plasmid. MBio. 2011; 2: e00204–1. 10.1128/mBio.00204-11 22045989 PMC3202755

[13] Dautzenberg MJD, Ossewaarde JM, de Kraker MEA, van der Zee A, van Burgh S, de Greeff SC, et al Successful control of a hospital-wide outbreak of OXA-48 producing Enterobacteriaceae in the Netherlands, 2009 to 2011. Euro Surveill. 2014; 19: pii = 20723. 10.2807/1560-7917.es2014.19.9.20723 24626209

[14] Thomson KS. Extended-spectrum-beta-lactamase, AmpC, and Carbapenemase issues. J Clin Microbiol. 2010; 48: 1019–1025. 10.1128/JCM.00219-10 20181902 PMC2849556

[15] Harris AD, Perencevich EN, Johnson JK, Paterson DL, Morris JG, Strauss SM, et al Patient-to-patient transmission is important in extended-spectrum beta-lactamase-producing Klebsiella pneumoniae acquisition. Clin Infect Dis. 2007; 45: 1347–1350. 17968833 10.1086/522657

[16] Harris AD, Kotetishvili M, Shurland S, Johnson JA, Morris G, Nemoy LL, et al How important is patient-to-patient transmission in extended-spectrum beta-lactamase Escherichia coli acquisition. Am J Infect Control. 2007; 35: 97–101. 17327188 10.1016/j.ajic.2006.09.011

[17] Hilty M, Betsch BY, Bogli-Stuber K, Heiniger N, Stadler M, Küffer M, et al Transmission Dynamics of Extended-Spectrum beta-lactamase-Producing Enterobacteriaceae in the Tertiary Care Hospital and the Household Setting. Clin Infect Dis. 2012; 55: 967–975. 10.1093/cid/cis581 22718774 PMC3436924

[18] Diekmann O, Heesterbeek H, Britton T. Mathematical Tools for Understanding Infectious Disease Dynamics. Princeton (NJ): Princeton University Press; 2012.

[19] Cooper BS, Medley GF, Stone SP, Kibbler CC, Cookson BD, Roberts JA, et al Methicillin-resistant Staphylococcus aureus in hospitals and the community: stealth dynamics and control catastrophes. Proc Natl Acad Sci USA. 2004; 101: 10223–10228. 15220470 10.1073/pnas.0401324101 PMC454191

[20] Philipsen KR, Bootsma MCJ, Leverstein-van Hall MA, Cohen Stuart J, Bonten MJM. Dynamics of extended-spectrum beta-lactamases in E.coli: a mathematical model. In: Philipsen KR. Nonlinear Stochastic Modelling of Antimicrobial resistance in Bacterial Populations. PhD Dissertation, Technical University of Denmark. 2010.

[21] Maasstad Ziekenhuis Rotterdam. Maatschappelijk Jaarverslag 2011. 2012; Dutch.

[22] Bonten MJM, Bootsma MCJ. Nosocomial Transmission: Methicillin-Resistant Staphylococcus aureus (MRSA) In: Krämer A, Kretzschmar M, Krickeberg K. Modern Infectious Disease Epidemiology. New York (NY): Springer; 2010.

[23] Poirel L, Walsh TR, Cuvillier V, Nordmann P. Multiplex PCR for detection of acquired carbapenemase genes. Diagn Microbiol Infect Dis. 2011; 70: 119–123. 10.1016/j.diagmicrobio.2010.12.002 21398074

[24] Kaase M, Szabados F, Wassill L, Gatermann SG. Detection of carbapenemases in Enterobacteriaceae by a commercial multiplex PCR. J Clin Microbiol. 2012; 50: 3115–3118. 10.1128/JCM.00991-12 22785190 PMC3421779

[25] Doyle D, Peirano G, Lascols C, Lloyd T, Church DL, Pitout JD. Laboratory detection of Enterobacteriaceae that produce carbapenemases. J Clin Microbiol. 2012; 50: 3877–3880. 10.1128/JCM.02117-12 22993175 PMC3503014

