# Supplementary material for: Within-Host and Population Transmission of bla OXA-48 in K. pneumoniae and E. coli
Source: PLoS One. 2015 Oct 20;10(10):e0140960. doi: 10.1371/journal.pone.0140960 (PMC4613826; doi:10.1371/journal.pone.0140960)
Supplement: S1 File — (PDF) [file pone.0140960.s001.pdf]

Supporting Information 1. Calculations.

<u>Differential equations</u>

The model used (Figure S1) can be described by the differential equations below. In the equations, *W* represents the number of uncolonized hospitalized patients (K- C-), *X* the number of hospitalized patients colonized with *E. coli*$_{\text{OXA-48}}$ (K- C+), *Y* the number of hospitalized patients colonized with *K. pneumoniae*$_{\text{OXA-48}}$ (K+ C-), and *Z* the number of hospitalized patients colonized with both (K+ C+). Since the hospital population size is constant, $Z = N – (W + X + Y)$.

Ah to *Dh* represent the same types of individuals in the community with a high risk of readmission: *Ah* represents the number of uncolonized individuals (K- C-), *Bh* the number of individuals colonized with *E. coli*$_{\text{OXA-48}}$ (K- C+), *Ch* the number of individuals colonized with *K. pneumoniae*$_{\text{OXA-48}}$ (K+ C-), and *Dh* the number of individuals colonized with both (K+ C+). *Al* to *Dl* represent the same types of individuals in the community with a low risk of readmission.

$$\frac{dW}{dt} = -\alpha W + \varphi_1 Ah + \varphi_2 Al - \beta_K \frac{Y+Z}{N} W - \beta_C \frac{X+Z}{N} W$$

$$\frac{dX}{dt} = -\alpha X + \varphi_1 Bh + \varphi_2 Bl + \beta_C \frac{X+Z}{N} W - \beta_K \frac{Y+Z}{N} X - \lambda_{CK} X$$

$$\frac{dY}{dt} = -\alpha Y + \varphi_1 Ch + \varphi_2 Cl + \beta_K \frac{Y+Z}{N} W - \beta_C \frac{X+Z}{N} Y - \lambda_{KC} Y$$

$$\frac{dAh}{dt} = \alpha W - \varphi_1 Ah - \chi Ah + \gamma_C Bh + \gamma_K Ch$$

$$\frac{dBh}{dt} = \alpha X - \varphi_1 Bh - \chi Bh + \gamma_K Dh - \gamma_C Bh - \lambda_{CK} Bh$$

$$\frac{dCh}{dt} = \alpha Y - \varphi_1 Ch - \chi Ch + \gamma_C Dh - \gamma_K Ch - \lambda_{KC} Ch$$

$$\frac{dDh}{dt} = \alpha Z - \varphi_1 Dh - \chi Dh - \gamma_K Dh - \gamma_C Dh + \lambda_{CK} Bh + \lambda_{KC} Ch$$

$$\frac{dAl}{dt} = \chi Ah - \varphi_2 Al + \gamma_C Bl + \gamma_K Cl$$

$$\frac{dBl}{dt} = \chi Bh - \varphi_2 Bl + \gamma_K Dl - \gamma_C Bl - \lambda_{CK} Bl$$

$$\frac{dCl}{dt} = \chi Ch - \varphi_2 Cl + \gamma_C Dl - \gamma_K Cl - \lambda_{KC} Cl$$

$$\frac{dDl}{dt} = \chi Dh - \varphi_2 Dl - \gamma_K Dl - \gamma_C Dl + \lambda_{CK} Bl + \lambda_{KC} Cl$$

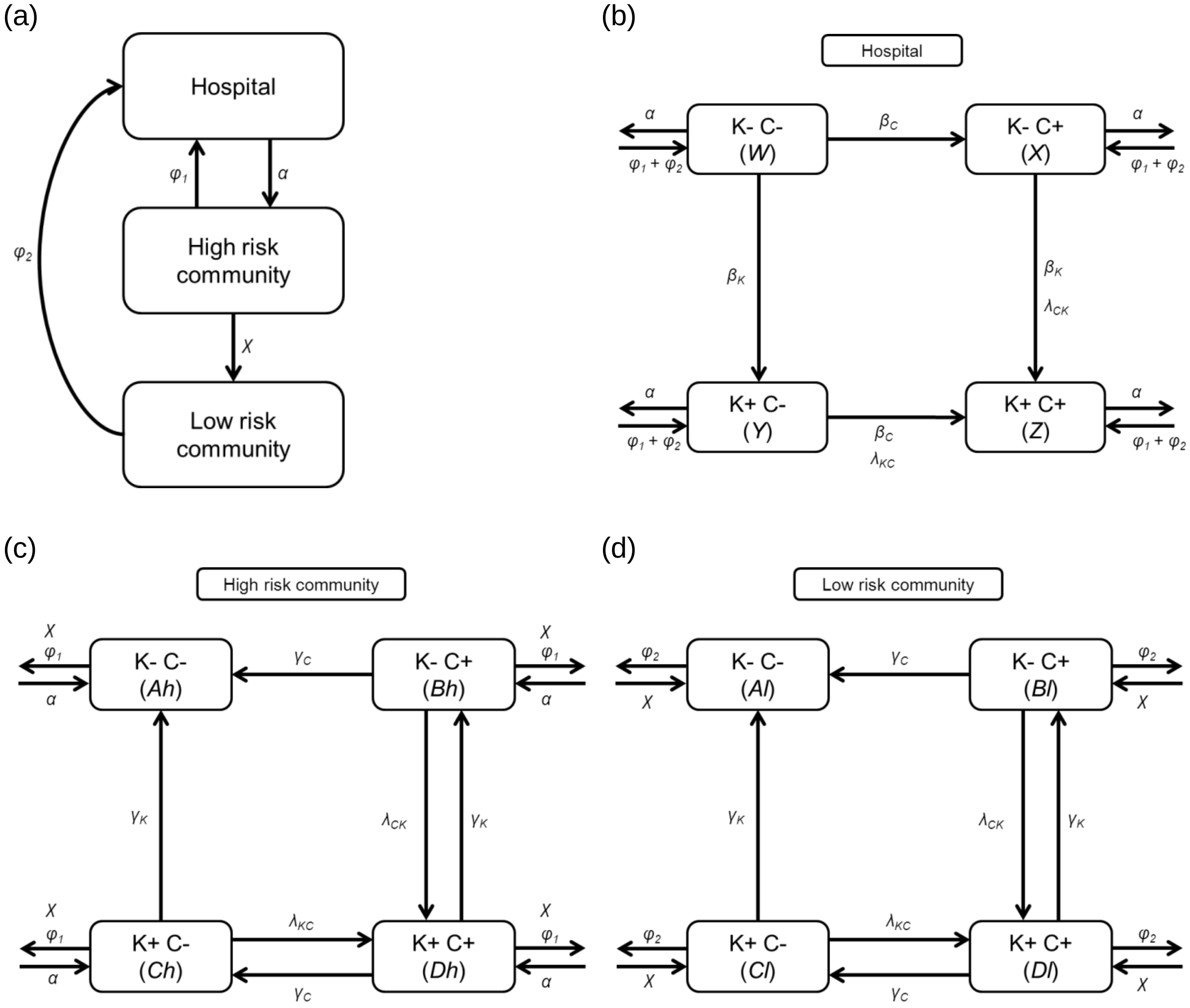

Figure S1. OXA-48 model.
(a) Model of population flow
(b) Within-host model in the hospital
(c) Within-host model in the community with a high risk of readmission
(d) Within-host model in the community with a low risk of readmission

K- C-: $\textit{bla}_{\text{OXA-48}}$ negative (both *K. pneumoniae* and *E. coli* are susceptible)
K- C+: $\textit{E. coli}_{\text{OXA-48}}$ (*K. pneumoniae* is susceptible)
K+ C-: $\textit{K. pneumoniae}_{\text{OXA-48}}$ (*E. coli* is susceptible)
K+ C+: $\textit{K. pneumoniae}_{\text{OXA-48}}$ and $\textit{E. coli}_{\text{OXA-48}}$

Calculation of R_0 and R_A

R_0 was calculated using the methodology described in the book of Diekmann et al. [18] First, the transition matrix Σ was determined. In Σ, all changes of states except for cross-transmissions are incorporated:

$$\begin{pmatrix} -\alpha-\lambda_{CK} & 0 & 0 & \varphi_1 & 0 & 0 & \varphi_2 & 0 & 0 \\ 0 & -\alpha-\lambda_{KC} & 0 & 0 & \varphi_1 & 0 & 0 & \varphi_2 & 0 \\ \lambda_{CK} & \lambda_{KC} & -\alpha & 0 & 0 & \varphi_1 & 0 & 0 & \varphi_2 \\ \alpha & 0 & 0 & -\gamma_C-\lambda_{CK}-\varphi_1-\chi & 0 & \gamma_K & 0 & 0 & 0 \\ 0 & \alpha & 0 & 0 & -\gamma_K-\lambda_{KC}-\varphi_1-\chi & \gamma_C & 0 & 0 & 0 \\ 0 & 0 & \alpha & \lambda_{CK} & \lambda_{KC} & -\gamma_C-\gamma_K-\varphi_1-\chi & 0 & 0 & 0 \\ 0 & 0 & 0 & \chi & 0 & 0 & -\gamma_C-\lambda_{CK}-\varphi_2 & 0 & \gamma_K \\ 0 & 0 & 0 & 0 & \chi & 0 & 0 & -\gamma_K-\lambda_{KC}-\varphi_2 & \gamma_C \\ 0 & 0 & 0 & 0 & 0 & \chi & \lambda_{CK} & \lambda_{KC} & -\gamma_C-\gamma_K-\varphi_2 \end{pmatrix}$$

Next, minus the inverse of Σ ($-\Sigma^{-1}$) is calculated. The elements of $-\Sigma^{-1}$ have a clear interpretation: the element $-(\Sigma^{-1})_{ij}$ is the expected time that an individual will spend in state *i*, given that it is currently in state *j*.

Thereafter, the next-generation matrix (NGM) can be calculated, using the cross-transmission parameters (β_K and β_C). Element *ij* of the NGM can be interpreted as the expected number of new colonizations starting in state *i*, caused by an infected individual in state *j*. Since a newly colonized person always starts his 'colonized life' in state *X* or *Y*, the NGM can be reduced to:

$$\begin{pmatrix} \beta_C *(-(\Sigma^{-1})_{11}+-(\Sigma^{-1})_{31}) & \beta_C *(-(\Sigma^{-1})_{12}+-(\Sigma^{-1})_{32}) \\ \beta_K *(-(\Sigma^{-1})_{21}+-(\Sigma^{-1})_{31}) & \beta_K *(-(\Sigma^{-1})_{22}+-(\Sigma^{-1})_{32}) \end{pmatrix}$$

R_0 is then the dominant eigenvalue of the NGM. An explicit expression for R_0 in terms of the model parameters does exist, but is too large to write down here.

For the calculation of R_A we only focused on the hospital dynamics (Figure S1B), ignoring readmissions. The transition matrix Σ is then:

$$\begin{pmatrix} -\alpha-\lambda_{CK} & 0 & 0 \\ 0 & -\alpha-\lambda_{KC} & 0 \\ \lambda_{CK} & \lambda_{KC} & -\alpha \end{pmatrix}$$

Using the same methodology as described above, the following NGM is obtained:

$$\begin{pmatrix} \frac{\beta_C}{\alpha} & \frac{\beta_C \lambda_{KC}}{\alpha^2+\alpha\lambda_{KC}} \\ \frac{\beta_K \lambda_{CK}}{\alpha^2+\alpha\lambda_{CK}} & \frac{\beta_K}{\alpha} \end{pmatrix}$$

Again, R_A is the dominant eigenvalue of this NGM.

Calculation colonization duration with HGT

In order to determine the influence of HGT on the duration of colonization with *K. pneumoniae*$_{OXA\text{-}48}$ or *E. coli*$_{OXA\text{-}48}$, we focused on the situation outside the hospital, where loss of colonization is possible (Figure S2). We will elaborate on the calculation of *K. pneumoniae*; the calculation for *E. coli* is analogous.

The mean duration of colonization with *K. pneumoniae*$_{OXA\text{-}48}$, as calculated from the data, is $1/\gamma_K$. If HGT is included, then an individual can 'start' being colonized in state K+C-, or in state K+C+. *T* is defined as the mean duration of colonization with *K. pneumoniae*$_{OXA\text{-}48}$ given a start in K+C- and *U* is defined as the mean duration of colonization with *K. pneumoniae*$_{OXA\text{-}48}$ given a start in K+C+. The following expressions can then be derived for *T* and *U*:

$$T = \frac{1}{\gamma_K + \lambda_{KC}} + \frac{\lambda_{KC}}{\gamma_K + \lambda_{KC}} U$$

$$U = \frac{1}{\gamma_K + \gamma_C} + \frac{\gamma_C}{\gamma_K + \gamma_C} T + \frac{\gamma_K}{\gamma_K + \gamma_C} \frac{\lambda_{CK}}{\gamma_C + \lambda_{CK}} U$$

Solving these equations for *T* and *U* gives the following expression for *T*:

$$T = \frac{\gamma_C{}^2 + \lambda_{CK}\lambda_{KC} + \gamma_C(\gamma_K + \lambda_{CK} + \lambda_{KC})}{\gamma_C \gamma_K (\gamma_C + \gamma_K + \lambda_{CK} + \lambda_{KC})}$$

The ratio of the duration of colonization with *K. pneumoniae*$_{OXA\text{-}48}$ with and without HGT can then be calculated as:

$$\frac{T}{1/\gamma_K} = \gamma_K T$$

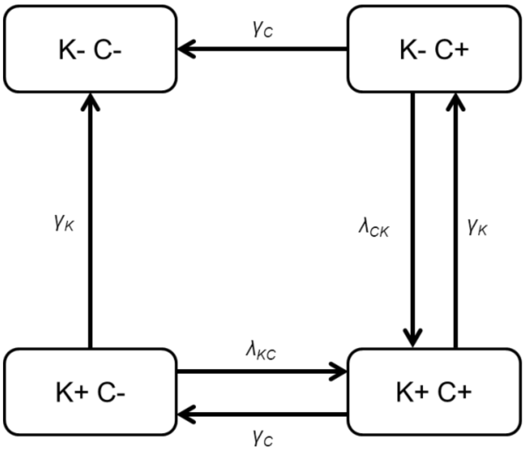

Figure S2. OXA-48 model (community).

Representation of the model used to calculate the influence of HGT on the duration of colonization.
