# Supplementary material for: Within-Host and Population Transmission of bla OXA-48 in K. pneumoniae and E. coli
Source: PLoS One. 2015 Oct 20;10(10):e0140960. doi: 10.1371/journal.pone.0140960 (PMC4613826; doi:10.1371/journal.pone.0140960)
Supplement: S2 File — (PDF) [file pone.0140960.s002.pdf]

Supporting Information 2. Parametric and non-parametric estimation of the survival curve.

In Figure S3 the survival curve of *K. pneumoniae*$_{OXA\text{-}48}$ is plotted, estimated using a non-parametric maximum likelihood method (blue line) and a assuming an exponential distribution of the survival curve (red line). The lines are not identical, but the differences are relatively small and therefore the exponential distribution seems like a good approximation of the non-parametric survival curve. The same applies to Figure S4, where the survival curve of *E. coli*$_{OXA\text{-}48}$ is shown. Since we also assumed an exponential distribution of the duration of colonization in our model, we used the parametric estimation of the survival curve.
Simultaneously with the survival curves, the sensitivity of the screening process was estimated using a maximum likelihood approach. We assumed that negative cultures in between positive cultures were false negative, thereby reducing the sensitivity.

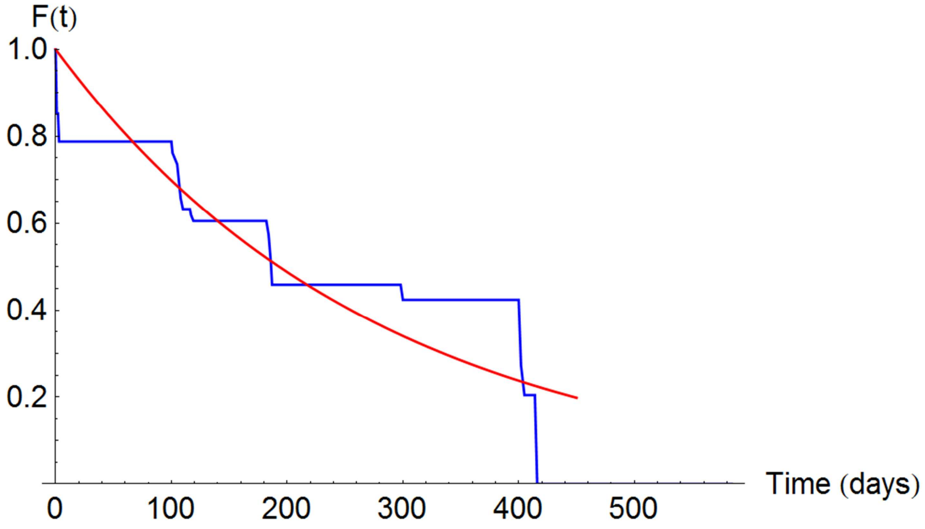

Figure S3. Survival curve of *K. pneumoniae*$_{OXA\text{-}48}$.
Parametric (red) and non-parametric (blue) estimation of the survival curve of *K. pneumoniae*$_{OXA\text{-}48}$.

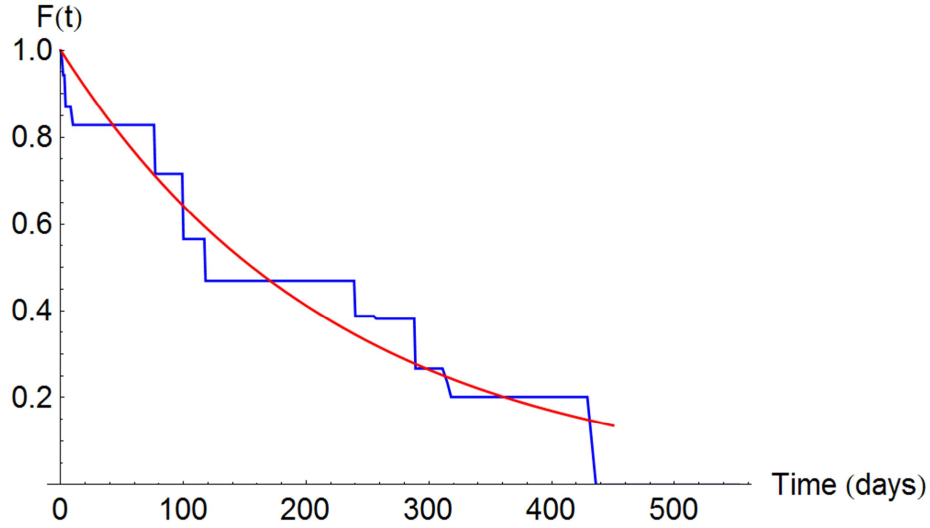

Figure S4. Survival curve of *E. coli*$_{\mathbf{OXA\text{-}48}}$.
Parametric (red) and non-parametric (blue) estimation of the survival curve of *E. coli*$_{OXA\text{-}48}$.
