# Supplementary material for: Within-Host and Population Transmission of bla OXA-48 in K. pneumoniae and E. coli
Source: PLoS One. 2015 Oct 20;10(10):e0140960. doi: 10.1371/journal.pone.0140960 (PMC4613826; doi:10.1371/journal.pone.0140960)
Supplement: S3 File — (PDF) [file pone.0140960.s003.pdf]

Supporting Information 3. Sensitivity analyses.

In Figure S5, both HGT-rates are varied with a factor X (blue line) and also the duration of colonization with *K. pneumoniae*$_{\text{OXA-48}}$ and *E. coli*$_{\text{OXA-48}}$ is varied with a factor X (red line). It is clear that increasing the HGT-rates increases the effect of *E. coli* on R_0 (e.g., expressed as the % change in R_0 when detecting *E. coli*$_{\text{OXA-48}}$ by PCR and hypothetically eradicating it). Although the estimates from the outbreak data leave some room for uncertainty, increasing the parameter values has a mild effect on the results. The effect of increasing the duration of colonization is stronger. An example of individuals who might have a longer duration of colonization could be immunocompromized patients.

In Figure S6, cross-transmission in the community is included. To assure that 80% of the transmission would take place in the hospital and keeping the ratio of β_K:β_C 3:1 both in the hospital and in the community, the cross-transmission parameters in the community should be 0.0050 times the values of the parameters in the hospital. β_K and β_C were chosen such that R_0 at the baseline values for the parameters was the same as in the rest of the analyses. The lines are steeper than in Figure 1 and so the influence of *E. coli*$_{\text{OXA-48}}$ on R_0 is bigger, but only slightly.

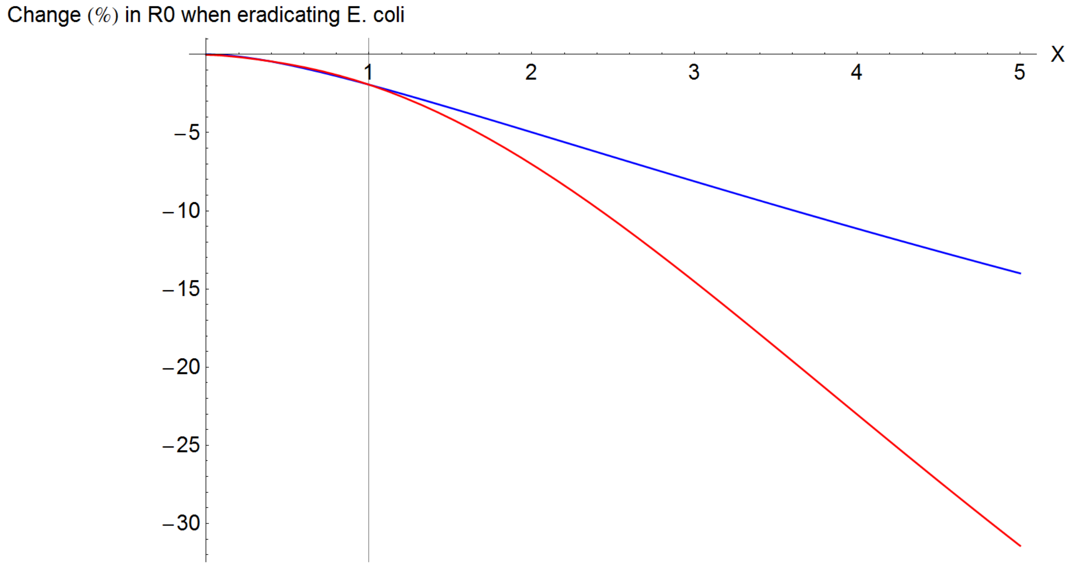

Figure S5. Results sensitivity analyses on λ_{KC} - λ_{CK} and γ_K - γ_C.
X is the factor with which λ_{KC} and λ_{CK} (blue line) and $1/\gamma_K$ and $1/\gamma_C$ (red line) are changed; X=1 is the original situation. All other parameters are kept constant.

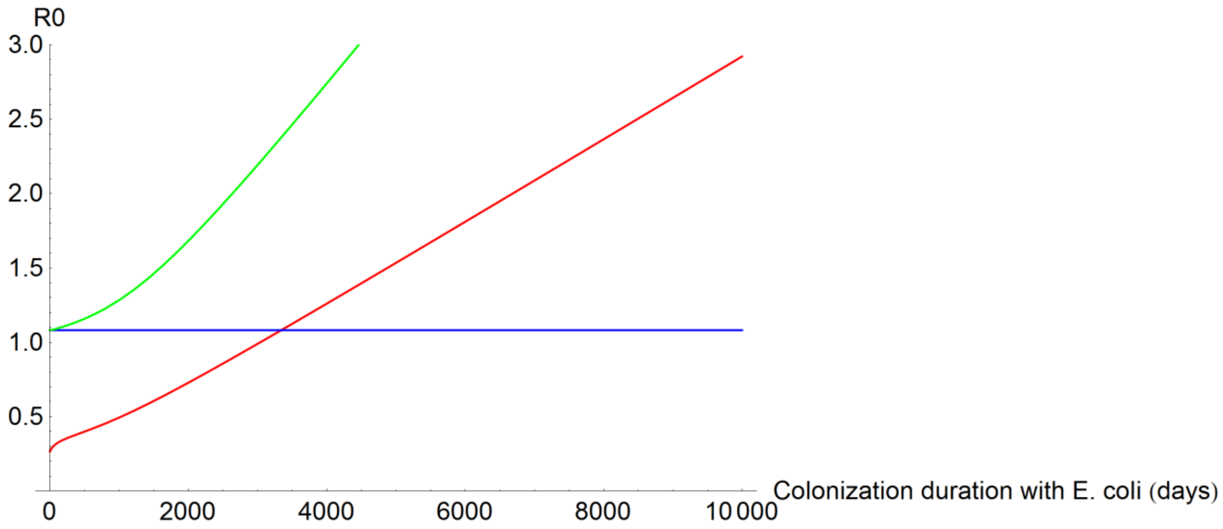

Figure S6. Influence of within-host horizontal gene transfer on R_0, including cross-transmission in the community.
The red line depicts the R_0 of *E. coli*$_{\text{OXA-48}}$ as a function of the duration of colonization with *E. coli*$_{\text{OXA-48}}$ when there is no HGT, the blue line represents the R_0 of *K. pneumoniae*$_{\text{OXA-48}}$ as a function of the duration of colonization with *E. coli*$_{\text{OXA-48}}$ when there is no HGT, and the green line represents R_0 as a function of the duration of colonization with *E. coli*$_{\text{OXA-48}}$ when there is HGT. R_0 is arbitrarily set at 1.1.
